# Enhanced Microwave Absorption and Electromagnetic Properties of Si-Modified rGO@Fe_3_O_4_/PVDF-*co*-HFP Composites

**DOI:** 10.3390/ma13040933

**Published:** 2020-02-20

**Authors:** Yuexuan Li, Yugang Duan, Chengmeng Wang

**Affiliations:** State Key Lab for Manufacturing Systems Engineering, School of Mechanical Engineering, Xi’an Jiaotong University, Xi’an 710054, China; liyuexuan@stu.xjtu.edu.cn (Y.L.); wangchengmeng@stu.xjtu.edu.cn (C.W.)

**Keywords:** absorbing materials, Si-modified rGO@Fe_3_O_4_/PVDF-*co*-HFP composites, dielectric loss, magnetic loss, electromagnetic properties

## Abstract

Graphene has been regarded as one of the most promising two-dimensional nanomaterials. Even so, graphene was still faced with several key issues such as impedance mismatching and narrow bandwidth, which have hindered the practical applications of graphene-based nanocomposites in the field of microwave absorption materials. Herein, a series of Si-modified rGO@Fe_3_O_4_ composites were investigated and fabricated by a simple method. On one hand, the degree of defects in graphene carbon could be tuned by different silane coupling reagents, which were beneficial to enhancing the dielectric loss. On the other hand, the spherical Fe_3_O_4_ nanoparticles provided the magnetic loss resonance, which contributed to controlling the impedance matching. Subsequently, the electromagnetic absorption (EMA) properties of Si-modified rGO@Fe_3_O_4_ composites with poly(vinylidene fluoride-*co*-hexafluoropropylene) (PVDF-*co*-HFP) were investigated in this work. As a result, the Si(2)-rGO@Fe_3_O_4_/PVDF-*co*-HFP composite exhibited the excellent EMA performance in the range of 2–18 GHz. The maximum reflection loss (*RL_max_*) reached −32.1 dB at 3.68 GHz at the thickness of 7 mm and the effective absorption frequency bandwidth for reflection loss (*RL*) below −10 dB was 4.8 GHz at the thickness of 2 mm. Furthermore, the enhanced absorption mechanism revealed that the high-efficiency absorption performance of Si(2)-rGO@Fe_3_O_4_/PVDF-*co*-HFP composite was attributed to the interference absorption (quarter-wave matching model) and the synergistic effects between Si(2)-rGO@Fe_3_O_4_ and PVDF-*co*-HFP. This work provides a potential strategy for the fabrication of the high-performance EMA materials.

## 1. Introduction

With the rapid propagation of the electronic devices—especially personal mobile phones, satellite communication, radar detectors, and other communication devices—electromagnetic waves (EMW), taken as “pollution”, are growing to be a huge threat to human health and has been attracted consideration attention [1]. Thus, great efforts have been devoted to exploiting the electromagnetic absorption (EMA) materials with low mass density, ultra-thin, and high EMA performance to eliminate the superfluous EMWs [2,3,4].

In the past decades, metal powders [5], graphite [6], and ferrites [7,8] were the mainstream materials in the field of the EMA materials. However, there are several drawbacks existed in these traditional EMA materials, such as high weight, narrow bandwidth, and unsatisfactory microwave absorption performance, have seriously hindered their practical applications. Nowadays, the carbon-based nanomaterials—such as multi-walled carbon nanotubes (MWCNTs), reduced graphene oxide (rGO), and carbon black—have been given highly attention in the field of aerospace engineering [9,10,11,12]. Particularly, rGO, as one of the most promising carbon-based nanomaterials, is commonly used as a high dielectric material. Because it possesses several excellent characteristics such as being lightweight, and having high specific surface area and abundant surface defects (such as diverse oxygen-containing groups) as well as its excellent mechanical properties. Even so, in terms of EMA impedance principle, pure rGO still is insufficient for impedance matching due to the limitation of single dielectric loss behavior [10,13,14]. Therefore, several scholars have focused on how to adjust the composition of nanomaterial to optimize their electromagnetic (EM) parameters, so as to achieve excellent microwave attenuation. For example, Jin et.al reported the chemical reduction process of rGO by ascorbic acid (VC). It was suggested that the degree of lattice defects in rGO could be tuned by the increase of VC addition, which could significantly enhance the EMA performance [15]. Meng’s group also reported that the nitrogen-doped rGO sheet exhibited the excellent EMA performance in the G band (5.6–8.2 GHz) and X band (8.2–12.4 GHz) due to the existence of the lattice defects in nitrogen-doped rGO [16]. Except for optimizing EM parameters by tuning the degree of defects in rGO, rGO combined with other magnetic loss materials, such as, CoNi [13], CoFe_2_O_4_ [17,18], FeCo [18], Fe_3_O_4_ [19], and Fe@Fe_3_O_4_ [20], is also a meaningful way to control the EMA performance. Particularly, when the magnetically controlled Fe_3_O_4_ nanoparticles are directly packaged on the rGO sheet, these results contribute to optimizing the impedance matching and enhance the EMW attenuation [21,22]. Random Fe_3_O_4_ nanoparticle packaging can be difficult to predict, thus making it difficult to obtain composite behavior at a large scale [23,24]. Meanwhile, this charge transfer between rGO and magnetic lossy materials can easily generate the interfacial polarization, dipole relaxation, and magnetic loss [25,26]. 

**Scheme 1:**
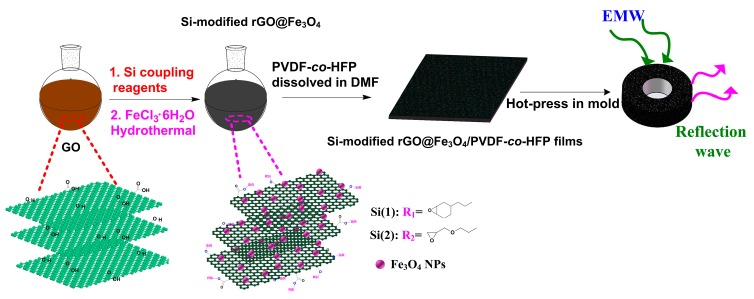
Schematic diagram of the synthesis procedure of Si-modified rGO@Fe_3_O_4_/PVDF-*co*-HFP composites.

In general, thermoplastic polymer matrices play an important role in the EMA composites due to their excellent mechanical properties [27,28]. Meanwhile, several interfacial effects or synergetic effects have been constantly occurred between the nano-fillers and thermoplastic polymer matrices, which are available for the enhancement of EMA performance of the absorbers [29,30]. Poly(vinylidene fluoride-*co*-hexafluoropropylene) (PVDF-*co*-HFP), as a typical dielectric loss material, has been widely applied in the various fields owning to its unique dielectric properties, high mechanical stability and excellent chemical resistance [31,32]. Herein, the fabrication process of two types of Si-modified rGO@Fe_3_O_4_/PVDF-*co*-HFP composites was investigated (Scheme 1). The EMA performance of Si-modified rGO@Fe_3_O_4_/PVDF-*co*-HFP composites could be tuned by different silane groups. Remarkably, the Si(2)-rGO@Fe_3_O_4_/PVDF-*co*-HFP composite achieved the maximum reflection loss (*RL_max_*) value of −32.1 dB (3.68 GHz) at the thickness of 7 mm and the effective absorption frequency bandwidth for reflection loss (*RL*) below −10 dB was 4.8 GHz at the thickness of 2 mm. Furthermore, the microwave absorption mechanism of Si-modified rGO@Fe_3_O_4_/PVDF-*co*-HFP has been illustrated in detail. Notably, the lattice defects in Si-modified rGO and the synergetic effects of PVDF-*co*-HFP play an important role in the enhancement of the EMA performance. The analysis of the interference absorption and the impedance matching can also preferably illustrate the high EMA performance of Si-modified rGO@Fe_3_O_4_/PVDF-*co*-HFP composites.

## 2. Materials and Methods 

### 2.1. Materials

Silane coupling reagents, including (3, 4-epoxycyclohexyl) ethyltrimethoxysilane {Si(1)} and 3-glycidoxypropyldimethoxymethylsilane {Si(2)}, were purchased from Energy Chemical Co., Ltd, Peking, China. Commercial graphene oxide (GO) (1000 mesh) was obtained from Beijing Carbon Century Technology Co., Ltd, Peking, China. Poly (vinylidene fluoride-*co*-hexafluoropropylene) (PVDF-*co*-HFP) was provided by Sigma-Aldrich Chemical Company (Sigma-Aldrich (Wuxi) Life Science&Tech. Co., Ltd., Jiangsu, China). Sodium acetate (NaOAc), ethylene glycol (EG), polyethylene glycol (PEG-200), and iron(III) chloride hexahydrate (FeCl_3_·6H_2_O) were purchased from Aladdin Chemical Reagent Co., Ltd, Shanghai, China. All chemical reagents were used as received without further purification.

### 2.2. Preparation of Si-Modified GO

Si-modified GO was fabricated by a silane coupling reagent according to references [33,34]. First, 200 mg GO suspension (1 mg·mL^−1^ in ethanol) was treated with hydrochloric solution (0.1 M) for pH = 3–4. Afterwards, 10 mL of silane coupling reagent was added into above mixed solution with continuously magnetic stirring at 60 °C for 24 h. After cooling to the room temperature and washed with water and ethanol three times, the Si-modified GO was obtained. The as-produced Si-modified GOs under Si(1) and Si(2) silane coupling reagent were denoted as Si(1)-GO and Si(2)-GO, respectively.

### 2.3. Preparation of Si-Modified rGO@Fe_3_O_4_ Nanocomposites

Si-modified rGO@Fe_3_O_4_ nanocomposites were synthesized by the hydrothermal reaction according to literature [35]. First, 90 mg as-obtained Si-modified GO and 900 mg FeCl_3_·6H_2_O were dispersed in 5 mL EG by ultrasonically treated for 0.5 h to obtain a homogeneous solution. Second, the above solution was slowly added into the 5 mL of NaOAc solution (0.2 g·mL^−1^ in EG) at 40 °C for 0.5 h. Third, the homogeneous mixture was transferred into a 50 mL Teflon lined autoclave and heated at 200 °C for 10 h. Finally, the as-prepared Si-modified rGO@Fe_3_O_4_ was separated with magnet and washed with ethanol and deionized water 5 times. After drying in 50 °C for 12 h, the Si-modified rGO@Fe_3_O_4_ was achieved. According to the silane coupling reagent used, the final products were donated as Si(1)-rGO@Fe_3_O_4_, Si(2)-rGO@Fe_3_O_4_, respectively. For comparison, rGO@Fe_3_O_4_ was also prepared in the existence of Fe^3+^ and NaOAc.

### 2.4. Fabrication of Si-Modified rGO@Fe_3_O_4_/PVDF-co-HFP Composites

PVDF-*co*-HFP was first ultrasonically dissolved in 5 mL *N, N’*-dimethylformamide (DMF) at 40 °C for 1 h. Subsequently, the different mass contents of as-obtained Si-modified rGO@Fe_3_O_4_ fillers were added into the above transparent solution and sonicated for 3 h. Finally, the mixture was dried in the vacuum oven at 120 °C for 3 h until the solvent evaporated completely. All samples were compressed into a hollow ring (*Φ_outer_:* 7.0 mm, *Φ_inner_:* 3.0 mm) by hot press at 200 °C for 10 min under 10 MPa and then cooling to room temperature naturally. Based on the different mass fractions of Si-modified rGO@Fe_3_O_4_ fillers used, the final products were labeled as Si(1)-rGO@Fe_3_O_4_/PVDF-*co*-HFP (S1-x wt %), Si(2)-rGO@Fe_3_O_4_/PVDF-*co*-HFP (S2-x wt %), respectively. x represents the mass fraction of fillers in the composites. For comparison purposes, rGO@Fe_3_O_4_/PVDF-*co*-HFP (S0-x wt %) was also fabricated.

### 2.5. Characterization

Powder X-ray diffraction (XRD) patterns of the samples were recorded on a Bruker D8 (Bruker AXS, Karlsruhe, Germany) advanced diffractometer using a Cu Kalpha radiation (λ = 0.15405 nm). The morphologies of the samples were investigated by scanning electron microscope (SEM, GeminiSEM 500, Carl Zeiss AG, Oberkochen, Germany) and transmission electron microscopy (TEM, Hitachi HT-7700 at 100 kV, Hitachi Limited, Tokyo, Japan). Fourier transform infrared spectroscopy (FT-IR) was acquired using a Nicolet iS10 FT-IR spectrometer (Thermo Fisher Scientific, Waltham, MA, USA). Raman spectroscopies were studied by a single monochromator with a microscope (Renishaw inVia, Renishaw plc, London, UK) equipped with CCD array detector (1024 × 256 pixels) and an edge filter and 633 nm. X-ray photoelectron spectroscopy (XPS) was measured with a Thermo Fisher Scientific ESCALAB Xi^+^ spectrometer (Thermo Fisher Scientific, Waltham, MA, USA) equipped with a monochromatic Al Kα radiation. The binding energies were calibrated using the C 1s peak at 284.4 eV. The relative complex EM parameters in the 2–18 GHz frequency were measured by a microwave vector network analyzer (N5227A, Agilent technologies, Santa Clara, CA, USA).

## 3. Results

### 3.1. Morphology and Structure

**Figure 1:**
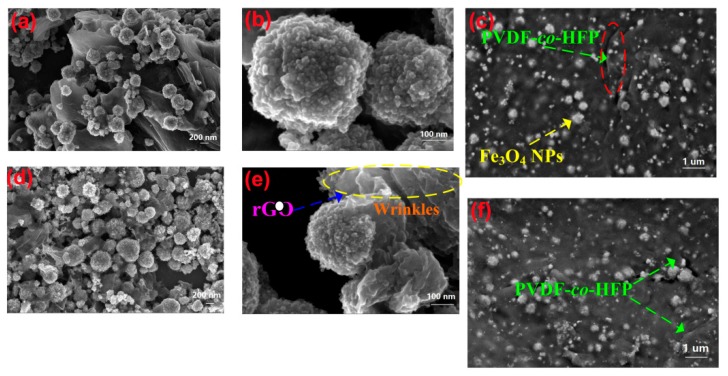
SEM images of (**a**,**b**) Si(1)-rGO@Fe_3_O_4_, (**c**) the film of S1 sample, (**d**,**e**) Si(2)-rGO@Fe_3_O_4_, (**f**) the film of S2 sample.

The SEM and TEM images of as-prepared samples are represented in Figure 1 and Figure S1 (Supplementary Materials). When the rGO was decorated by silane coupling reagents, there were more Fe_3_O_4_ nanoparticles (NPs) loaded on the surface of Si-modified rGO (Figure 1a,d). The reason is that the existence of –SiOH groups derives from the hydrolysis of –SiOCH_3_ group within the silane coupling reagent [36], which are preferable to coordinate with Fe^3+^. Then the Fe_3_O_4_ NPs were prepared by in situ reduction. Meanwhile, the spherical Fe_3_O_4_ NPs on the Si-modified rGO sheets are featured with rough surface and non-uniform diameters (Figure 1a,b,d,e). As demonstrated in Figure S1 (Supplementary Materials), the composite Si-modified rGO@Fe_3_O_4_ still possesses abundant wrinkles as same as that of rGO@Fe_3_O_4_ composite, which will benefit for improving the multiple-reflection of EMW [37,38,39]. Furthermore, the SEM images of the Si-modified rGO@Fe_3_O_4_/PVDF-*co*-HFP films illustrate that the Si-modified rGO@Fe_3_O_4_ are well dispersive in PVDF-*co*-HFP (Figure 1c,f).

**Figure 2:**
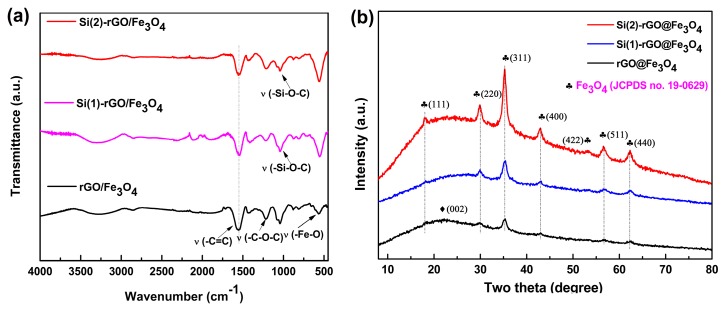
FT-IR spectra (**a**) and XRD patterns (**b**) of Si-modified rGO@Fe_3_O_4_ nanocomposites.

The analysis of FT-IR and XRD is first devoted to investigating the composition of the Si-modified rGO@Fe_3_O_4_ nanocomposites. In most cases, the bands at 1722 cm^−1^, 1617 cm^−1^, 1223 cm^−1^, 1052 cm^−1^, and 3356 cm^−1^ are attributed to C=O, C=C, C–O–C, C–OH, and –OH groups in the pure GO, respectively (Figure S2, Supplementary Materials). After modification with silane groups, the stretching vibration of –OH groups decrease obviously and the new peaks at 2918 cm^−1^ and 2840 cm^−1^ are observed, corresponding to the vibration of –CH_2_, –CH_3_ bond in silane groups. The peaks at about 1037 cm^−1^ is connected to the Si–O–C (Si–O–Si) bonds (Figure S2, Supplementary Materials), verifying the successful synthesis of Si-modified GO. After in situ hydrothermal reduction, the characteristic peak at 1543 cm^−1^ belongs to the vibration of C=C bond, suggesting that the Si-modified GO has been successfully reduced to the Si-modified rGO (Figure 2a). Moreover, a new peak at 556 cm^−1^ is characterized to the stretching vibration of –Fe–O bond in Fe_3_O_4_ NPs (Figure 2a). In order to investigate the crystalline structures of as-achieved Si-modified rGO@Fe_3_O_4_, the XRD patterns of all the samples are shown in Figure 2b. The broad peak at 2θ = 21.3° is attributed to the (002) facet of rGO [34]. After various silane groups are grafted on the surface of rGO, the diffraction peaks shift to a higher degree around 2θ = 25.1° {Si(1)} and 22.5° {Si(2)}, suggesting that the insertion of the silane group into GO can result in shrinking the *d*-spacing of the rGO sheet according to the Bragg equation [34]. At the same time, the seven obvious peaks at 18.1°, 30.1°, 35.4°, 43.1°, 53.5°, 56.8°, and 62.5° are assigned to (111), (220), (311), (400), (422), (511), and (440) planes of Fe_3_O_4_ NPs (JCPDS file no.19-0629), verifying that the composites contain the phase of Fe_3_O_4_ NPs.

**Figure 3:**
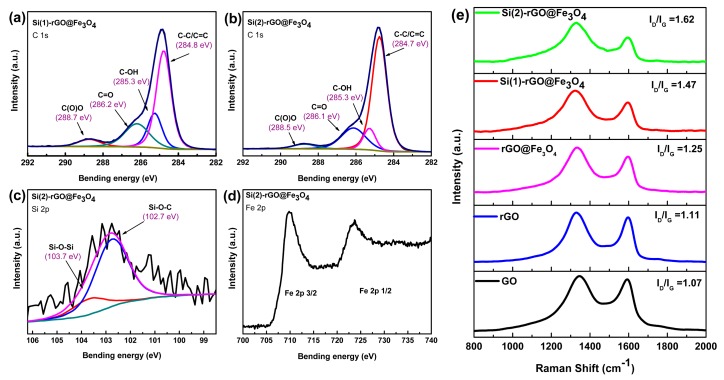
XPS spectra of Si-modified rGO@Fe_3_O_4_: High-resolution C1s spectra of (**a**) Si(1)-rGO@Fe_3_O_4_, (**b**)Si(2)-rGO@Fe_3_O_4_, (**c**) high resolution Si2p spectrum of Si(2)-rGO@Fe_3_O_4_, and (**d**) Fe2p spectrum of Si(2)-rGO@Fe_3_O_4_. (**e**) Raman spectra of all the samples.

To further confirm whether the Fe_3_O_4_ NPs is the unique magnetic phase in Si-modified rGO@Fe_3_O_4_ nanocomposites, XPS analysis of Fe 2p spectrum is illustrated in Figure 3d. Remarkably, two peaks at 711.5 eV and 725.1 eV is characterized to the Fe 2p 1/2 and Fe 2p 3/2, respectively, and no satellite peak at 719.0 eV indicates no γ-Fe_2_O_3_ phase in composites [40,41].

Besides, the XPS analysis also provides an avenue to better understand the chemical valence state of Si-modified rGO@Fe_3_O_4_ nanocomposites. For Si-modified GO, there have three elements corresponding to the C, O, and Si elements in the wide span spectra of Si-modified GO (Figure S3a, Supplementary Materials). The four peaks at 284.4 eV, 285.2 eV, 287.2 eV, and 289.3 eV are attributed to the C–C, C–OH, C=O, and C(O)O bonds of GO, respectively (Figure S3b, Supplementary Materials). After modification with silane groups, the intensity of C–C peak of Si(1)-GO obviously increases, whereas its C–OH (285.6 eV) and C=O (287.3 eV) peaks decrease remarkably (Figure S3c, Supplementary Materials). Compared with Si(1)-GO, the intensity of C=O peak of Si(2)-GO is greater than that of Si(1)-GO (Figure S3d, Supplementary Materials), suggesting that the Si(1) is more prone to coupling reaction with –COOH groups in GO. From the Si 2p spectrum of Si(2)-GO (Figure S3e, Supplementary Materials), the peaks at 102.6 eV and 103.5 eV stand for the Si–O–C and Si–O–Si bond, respectively, verifying that most Si(2) reagents have been grafted on the surface of GO and only a part of Si–O–Si groups were obtained by the self-condensation of Si(2) silane coupling reagent [36]. After hydrothermal reduction, there are several oxygen-containing groups still existed in the Si-modified rGO@Fe_3_O_4_ composite (Figure 3a,b). Notably, the intensity of C–OH peak of Si(2)-rGO@Fe_3_O_4_ is less than that of Si(1)-rGO@Fe_3_O_4_. This result reveals that the Si(2)-rGO@Fe_3_O_4_ possesses higher reduction degree, which is beneficial for improving the imaginary permittivity of the microwave absorption materials [15]. As shown in Figure 3c, the Si 2p spectrum of Si(2)-rGO@Fe_3_O_4_ exhibits the presence of residual –Si–O–C bonds and –Si–O–Si bonds, confirming that several Si(2) silane groups have been existed in Si(2)-rGO@Fe_3_O_4_ composite.

It is well known that the lattice defects in the graphene structure are beneficial for enhancing the EMA performance [19]. Here, the influence of various silane groups on rGO defects was investigated by Raman spectroscopy (Figure 3e). Generally, the D-band (1330 cm^−1^) and G-band (1595 cm^−1^) represent the lattice defects and the radical C–C stretching vibration of carbon sp^2^ hybrid in the graphene structure, respectively. The graphitization degree is typically determined by the intensity ratio *I_D_/I_G_* [42]. The higher the *I_D_/I_G_* value, the greater the lattice defects in graphene structure. As revealed in Figure 3e, the ratio *I_D_/I_G_* of rGO@Fe_3_O_4_ (1.25) is greater than that of rGO (1.11) due to the introduction of the Fe_3_O_4_ NPs. Furthermore, the *I_D_/I_G_* values of Si(1)-rGO@Fe_3_O_4_ and Si(2)-rGO@Fe_3_O_4_ are 1.47 and 1.62, respectively, implying that the silane group Si(2) grafted on the rGO surface contributes to generating abundant lattice defects in graphene structure.

### 3.2. Electromagnetic Properties

**Figure 4:**
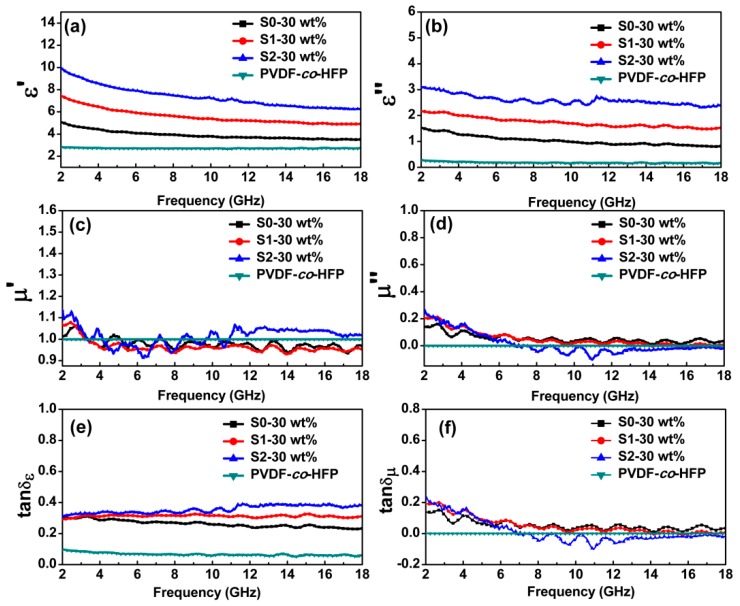
Frequency dependence of the EM parameters of all the samples: (**a**) real permittivity, (**b**) imaginary permittivity, (**c**) real permeability, (**d**) imaginary permeability, (**e**) dielectric loss, and (**f**) magnetic loss.

In most cases, the complex permittivity (*ε’* and *ε’’*) and relative permeability (*μ’* and *μ’’*) are two important EM parameters to judge the quality of the microwave absorption performance. In order to obtain the optimal mass fraction of Si-modified rGO@Fe_3_O_4_ in PVDF-*co*-HFP, the EMW properties of all the samples with different mass fractions loading were investigated in Figure S4–6 (Supplementary Materials). As a result, the Si-modified rGO@Fe_3_O_4_ powders with 30 wt % contents in PVDF-*co*-HFP are the best choice. As shown in Figure 4a,b, the pure PVDF-*co*-HFP has poor permittivity properties. After addition of fillers, the permittivity values of samples have obvious improved, especially, Si-modified rGO@Fe_3_O_4_/PVDF-*co*-HFP composites possess higher dielectric behavior. The *ε’* values of S1 is around 8, and its *ε’’* values reach around 2. Similarly, the *ε’* values of S2 become around 10 and its *ε’’* values are around 3. Based on the free electron theory, *ε’’∞σ/2πε_0_f*, where *σ* stands for the conductivity, it can be found that the S2 sample possesses the higher electrical conductivity, which is attributed to the induced dipoles of the existence of C–O, Si–O species on the surface of S2 composite or vibrations of atoms, ions, or electrons in the S2 composite. Furthermore, the real permeability (*μ’*) values of S1 and S2 samples are 1.07–0.95 and 1.13–1.01, respectively, which are higher than that of S0 sample without modified by silane groups (about 1.01–0.96) (Figure 4c). Generally, the imaginary permeability (*μ’’*) values of composites must be more than 0 [43]. As illustrated in Figure 4d, S2 sample is an exception, its *μ’’* values show negative in the range of 7–18 GHz frequency. The reason is that the negative *μ’’* is attributed to the radiation of the magnetic energy [43]. According to the Maxwell equations [44], the charges transferred in variable electric field is easy to produce the induced magnetic field, which may result in the formation of the magnetic energy [45].

As illustrated in Figure 4e, compared to the dielectric loss tangent (*tanδ_ε_ = ε”/ε’*) of the pure PVDF-*co*-HFP (*tanδ_ε_* = 0.1) and S0 sample (*tanδ_ε_* = 0.3), the higher *tanδ_ε_* values of the Si-modified rGO@Fe_3_O_4_/PVDF-*co*-HFP composites confirm that the silane groups grafted on the rGO surface are more favourable to increase the complex dielectric loss. In particular, S2 sample has the highest *tanδ_ε_* values, corresponding to 0.31–0.38. Furthermore, several resonance peaks in the complex *tanδ_ε_* indicate that the polarization effects may exist in the composites [46]. As shown in Figure 4f, the magnetic loss of samples in the range of 2–13 GHz are greater than that in the range of 13–18 GHz. However, the complex magnetic loss covers weaker values contrast to the dielectric loss. These results suggest that the EMA performance of Si-modified rGO@Fe_3_O_4_/PVDF-*co*-HFP composites is mainly attributed to dielectric loss rather than magnetic loss.

**Figure 5:**
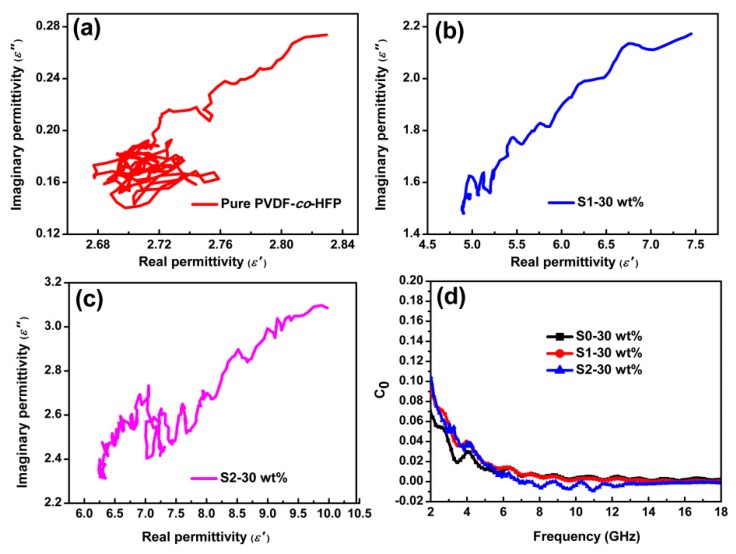
Cole-cole (**a**–**c**) and *C_0_* (**d**) curves for all the samples.

In a general way, the polarization effects of graphene-based composite mainly result from the lattice defects in graphene structure, dipole polarization, or interfacial polarization between rGO and other magnetic metal oxides and polymer matrix, which has an important influence on the complex EMA performance. According to the Cole–Cole semicircle principle, the complex relaxation process can be obtained via Equation (1) [47].

(1) $${\left(\varepsilon {}^{\prime }-\frac{\varepsilon_{s}+\varepsilon_{\infty }}{2}\right)}^{2}+{\left(\varepsilon {}^{\prime \prime }\right)}^{2}={\left(\frac{\varepsilon_{s}-\varepsilon_{\infty }}{2}\right)}^{2}$$

where *ε’* and *ε^∞^* refer to the static dielectric constant and the relative permittivity at boundless frequency, respectively. If Debye relaxation process is occurred in composites, several single semicircles are exhibited in the curve of *ε’* and *ε”* [47,48]. As illustrated in Figure 5a–c, the *ε’-ε”* plots of pure PVDF-*co*-HFP is mostly disordered, whereas there is plenty of semicircles in the curve of the samples modified by silane groups, verifying that the dielectric loss of Si-modified rGO@Fe_3_O_4_/PVDF-*co*-HFP composites may result from other multiple interfacial polarizations, including Si-modified rGO@Fe_3_O_4_, Fe_3_O_4_/PVDF-*co*-HFP and Si-modified rGO/PVDF-*co*-HFP interfaces, rather than Debye relaxation [22,49]. Besides, the lattice defects and the dipole polarization in Si-modified rGO@Fe_3_O_4_ fillers is also helpful for enhancing the EMA performance of samples decorated by silane groups owning to the existence of C–O, Si–O species on the surface of Si-modified rGO@Fe_3_O_4_ composites.

In most cases, the complex magnetic loss usually results from natural resonance, eddy-current effect, and domain-wall displacement [50]. The domain-wall displacement mainly exists in the 1–100 MHz [25,51], suggesting that the EMA performance of Si-modified rGO@Fe_3_O_4_/PVDF-*co*-HFP composites over the range of 2–18 GHz is not attributed to the domain-wall displacement. For the eddy current effect, it can be calculated by Equation (2) [1,52]

(2) $$C_{0}=\mu {}^{\prime \prime }{\left(\mu {}^{\prime }\right)}^{-2}f^{-1}$$

If the *C_0_* values are equal to the constant in the 2–18 GHz frequency, the complex magnetic loss only arises from the eddy-current [53]. As shown in Figure 5d, the *C_0_* values of all the samples vary with frequency, indicating that the complex magnetic loss behavior over the range of 2–18 GHz frequency is mainly attributed to the natural resonance [53,54].

On the basis of the transmission line theory, the EMA performance of Si-modified rGO@Fe_3_O_4_/PVDF-*co*-HFP composites was illustrated by the *RL* values, which were calculated by the following equations [47,55,56],

(3) $$Z_{in}=Z_{0}\sqrt{\frac{\mu_{r}}{\varepsilon_{r}}}\tanh\left[j(\frac{2\pi fd}{c})\sqrt{\mu_{r}\varepsilon_{r}}\right]$$

(4) $$RL=20\log\left|\frac{Z_{in}-Z_{0}}{Z_{in}+Z_{0}}\right|$$

where *Z_in_* refers to the normalized input impedance, *Z_0_* belongs to the impedance in free space (*Z_0_* = 377 Ω), *ε_r_* and *μ_r_* are the complex permittivity and relative permeability, respectively, *f* is the microwave frequency, *d* is the thickness of the composite, and *c* is the velocity of light.

**Figure 6:**
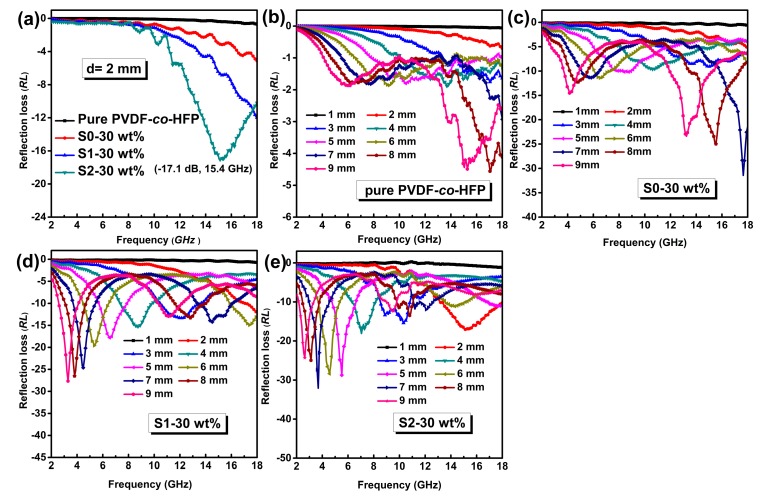
Frequency dependence of the calculated *RL* values of all the samples in the range of 2–18 GHz: (**a**) the thickness of 2 mm, (**b**–**e**) at different thickness.

As exhibited in Figure 6a, the pure PVDF-*co*-HFP at the thickness of 2 mm exhibits poor *RL* values (RL < −1 dB). After modification with silane groups, the Si-modified rGO@Fe_3_O_4_/PVDF-*co*-HFP composites exhibit the enhanced EMA ability in the range of 2–18 GHz, particularly, the S2 sample modified by Si(2) groups shows high-performance microwave absorption performance in 2–18 GHz frequency. The maximum reflection loss (*RL_max_*) values for S2 sample over the whole frequency range are −17.1 dB at 15.4 GHz. In addition, the S1 sample exhibits the electromagnetic absorption performance over the 18 GHz. Moreover, as shown in Figure 6b–e, the calculated *RL* values of samples decorated by silane groups in the range of 2–18 GHz are higher than that of pure PVDF-*co*-HFP and S0 samples without modified by silane groups. The *RL_max_* values for S1 and S2 samples are −27.7 dB at 3.3 GHz with d = 9 mm and −32.1 dB at 3.68 GHz with d = 7 mm, respectively. Meanwhile, S2 sample exhibits a wide absorption frequency bandwidth of 4.8 GHz (RL < −10 dB) at d = 2 mm, demonstrating that Si(2) silane coupling agent is beneficial to improving the EMW absorption broadband of the absorber.

To judge the high-efficiency EMW absorption properties of Si-modified rGO@Fe_3_O_4_/PVDF-*co*-HFP composite in the range of 2–18 GHz, the attenuation constant *α*, as one of influence factors, can be described by [23,57]

(5) $$\alpha =\frac{\sqrt{2}\pi f}{c}\times \sqrt{\left(\mu {}^{\prime \prime }\varepsilon {}^{\prime \prime }-\mu {}^{\prime }\varepsilon {}^{\prime }\right)+\sqrt{{\left(\mu {}^{\prime }\varepsilon {}^{\prime \prime }+\mu {}^{\prime \prime }\varepsilon {}^{\prime }\right)}^{2}+{\left(\mu {}^{\prime \prime }\varepsilon {}^{\prime \prime }-\mu {}^{\prime }\varepsilon {}^{\prime }\right)}^{2}}}$$

where *f* refers to the microwave frequency and *c* refers to the velocity of light. As illustrated in Figure S7 (Supplementary Materials), the S2 sample has the largest attenuation constant values in all the samples in the range of 2–18 GHz, suggesting that the microwave absorption performance of S2 samples is superior to that of S0 and S1 samples over the range of 2–18 GHz. That result is in good agreement with the Figure 6a. Except for the high *α* values, the impedance matching also plays a significant role in the enhanced microwave attenuation. Here, the modulus of the relative impedances *Z = |Z_in_/Z_0_|* have been calculated by the following Equations (3). When the *Z* value is almost equal to 1, meaning that the incident EMW can be completely inside the absorber, and then converted into other forms of energy, such as thermal energy [21,58]. As described in Figure S8 (Supplementary Materials), after decoration with silane coupling agent, the *Z* values of S1 and S2 samples are less than that of S0 sample. Particularly, the *Z* values in the S2 sample are closer to 1 in the range of 2.6–4 GHz, confirming that the S2 sample exhibits the high-efficiency absorption performance at the thickness of 7–9 mm.

In addition, a quarter-wavelength theory also has been further supplied to explain the EMW absorption principle, the relationship between the thickness (*t_m_*) and the relative frequency (*f_m_*) can be characterized by equation [47,59,60],

(6) $$t_{m}=nc/\left(4f_{m}{\left(\left|\varepsilon_{r}\right|\left|\mu_{r}\right|\right)}^{1/2}\right);n=1,3,5,\ldots \ldots$$

where |*ε_r_*| and |*μ_r_*| refers to the modulus of the complex permittivity and relative permeability, respectively. As illustrated in Figure S8 (Supplementary Materials), the black line is the calculated matching thickness (*t_m_^fit^*) and the red spots denote the experimental matching thickness (*t_m_^exp^*). For S2 sample, the values of *t_m_^exp^* almost coincide with that of the *t_m_^fit^* (n = 1), explaining that the excellent EMW performance of S2 sample is mainly attributed to the interference absorption.

**Scheme 2:**
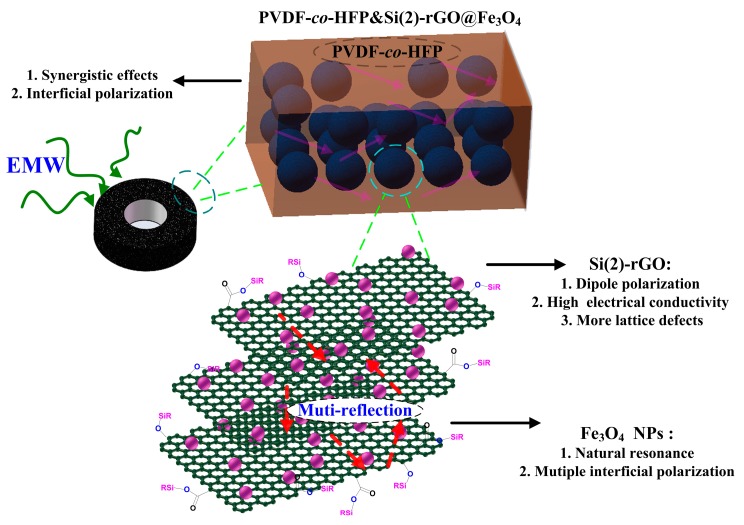
Schematic illustration of the proposed EMA mechanism of S2 sample.

Based on the above-mentioned analysis, we have proposed EMA mechanism to explain the reason why S2 sample possesses the high-efficiency absorption ability (Scheme 2). First, after modification with Si(2) silane coupling reagent, there are several dipole polarization in Si(2)-rGO@Fe_3_O_4_ fillers owning to the existence of C–O, Si–O group, which is helpful for enhancing the dielectric loss performance (Figure 5c) [14]. Moreover, based on imaginary permittivity analysis of S2-30wt % sample (Figure 4b), the high conductivity results from the Si(2) silane coupling grafted on the surface of rGO. Next, the dielectric loss of S2 sample is also attributed to the complex interfacial polarization (Si(2)-rGO&PVDF-*co*-HFP, Si(2)-rGO&Fe_3_O_4_, and Fe_3_O_4_&PVDF-*co*-HFP) and the lattice defects in graphene structure (Figure 3e), which is beneficial for the EM energy dissipation [16,40]. Meanwhile, the abundant of wrinkles in Si(2)-rGO would provide a channel for improving the multiple-reflection of EMW (Figure S1b,c, Supplementary Materials). Third, permeability parameters (*μ’* and *μ’’*) and *C_o_* curve indicated that magnetic loss effect of Fe_3_O_4_ NPs in S2 sample mainly resulted from the natural resonance in the range of 2–18 GHz (Figure 5d & Figure S9c, Supplementary Materials). Finally, the synergistic effect of Si(2)-rGO@Fe_3_O_4_ and PVDF-*co*-HFP was beneficial to further improving the microwave absorption properties of S2 sample (Figure 4 & Figure S9, Supplementary Materials), and the absorption interference dominates an important position in the high-efficiency microwave absorption performance of S2 sample (Figure S8, Supplementary Materials).

Moreover, Table 1 also gives the comparison of microwave absorption performance between those in the literature and the obtained Si-modified-rGO@Fe_3_O_4_/PVDF-*co*-HFP, it is clear from the Table 1 that the prepared Si-modified-rGO@Fe_3_O_4_/PVDF-*co*-HFP in this work exhibits primary advantages by fillers loading, effective bandwidth frequency, and effective thickness.

## 4. Conclusions

In total, a facile method to fabricate series of Si-modified rGO@Fe_3_O_4_/PVDF-*co*-HFP composites was investigated. Meanwhile, the EMW absorption performance of samples illustrated that the lattice defects in graphene modified by silane coupling reagents have a significant influence on the optimized EMA performance. Notably, the optimal Si(2)-rGO@Fe_3_O_4_/PVDF-*co*-HFP with 30 wt % loading (S2-30 wt %) exhibits the high-performance EMA ability. The *RL_max_* value of S2-30 wt % sample over the whole frequency range reaches −32.1 dB at 3.68 GHz at d = 7 mm and its maximum absorption bandwidth for RL < −10 dB is 4.8 GHz (13.2–18 GHz) at a thickness of 2 mm. On the basis of the proposed microwave attenuation mechanism, the high-efficiency EMA performance of S2-30 wt % sample mainly achieves from the interference absorption. Moreover, experimental results highlight that the interfacial polarization, dipole polarization and the higher degree of lattice defects in S2-30 wt % sample and synergistic effect of PVDF-*co*-HFP is beneficial to improving the dielectric loss. Simultaneously, its magnetic loss mainly originates the natural resonance effects of the Fe_3_O_4_ NPs, contributing to achieving the impedance matching. It is undoubtable that construction of Si(2)-rGO@Fe_3_O_4_/PVDF-*co*-HFP composite could provide new possibility and strategy for the fabrication of the high-performance broadband EMA materials.

## Figures and Tables

**Table 1:** Comparison of microwave absorption performance of similar composite in the range of 2–18 GHz

Samples	Loading (wt %)	RL_max_ (dB)	Thickness (mm)	EBD^a^ (GHz)	Ref.
Ni/PVDF	20	−42.08	3	2.5	[61]
MoS_2_/PVDF	25	−26.11	2.5	3.44	[62]
Mesoporous Fe_3_O_4_/C	40	−18.0	2.0	2.0	[63]
Graphene/ZnO	50	−45.1	2.2	2.5	[64]
PEDOT/RGO/Co_3_O_4_	50	−51.1	2.0	3.1	[65]
[Y_2_(MH)_6_]_n_·DMF	20	−22.78	5.0	2.24	[66]
Si(2)-rGO@Fe_3_O_4_/PVDF-*co*-HFP	30	−17.1	2.0	4.8	This work
Si(2)-rGO@Fe_3_O_4_/PVDF-*co*-HFP	30	−32.1	7.0	2.88	This work

^a^EBD: Effective bandwidth (RL < −10 dB).

## Supplementary Materials

The following are available online at https://www.mdpi.com/1996-1944/13/4/933/s1, Detailed TEM images of Si-modified rGO@Fe_3_O_4_ (Figure S1); FT-IR spectra (Figure S2) and XPS patterns (Figure S3) of Si-modified GO, relative complex (S0–S2) electromagnetic patterns with different mass fraction loading (Figure S4–S6) and complex attenuation constants (Figure S7); *|Z|* modulus (Figure S8) of Si-modified rGO@Fe_3_O_4_/PVDF-*co*-HFP with 30 wt% loading; and frequency dependence of the electromagnetic parameters of the Si(2)- rGO@Fe_3_O_4_/paraffin wax composite (Figure S9).
