# Supplementary material for: Enhanced Microwave Absorption and Electromagnetic Properties of Si-Modified rGO@Fe3O4/PVDF-co-HFP Composites
Source: Materials (Basel). 2020 Feb 20;13(4):933. doi: 10.3390/ma13040933 (PMC7079640; doi:10.3390/ma13040933)
Supplement: Supplementary file 1 [file materials-13-00933-s001.pdf]

materials

Supplementary Materials

Enhanced Microwave Absorption and Electromagnetic Properties of Si-Modified rGO@Fe_3O_4/PVDF-*co*-HFP Composites

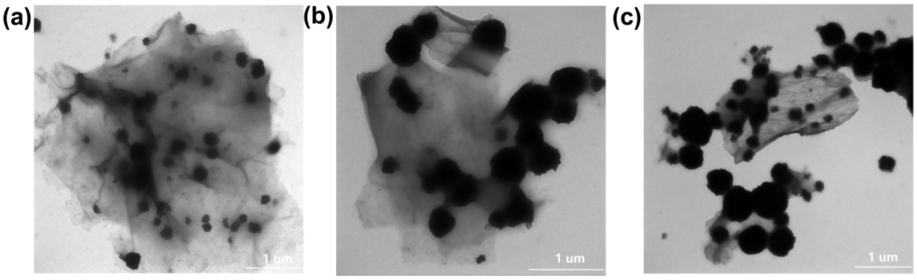

Figure S1. TEM images of (**a**) rGO@Fe_3O_4, (**b**) Si(1)-rGO@Fe_3O_4 and (**c**) Si(2)-rGO@Fe_3O_4 nanocomposites.

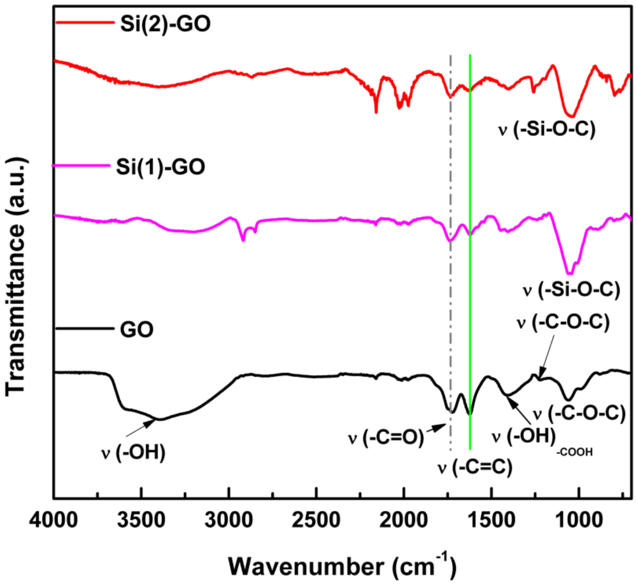

Figure S2. FT-IR spectra of Si-modified GO.

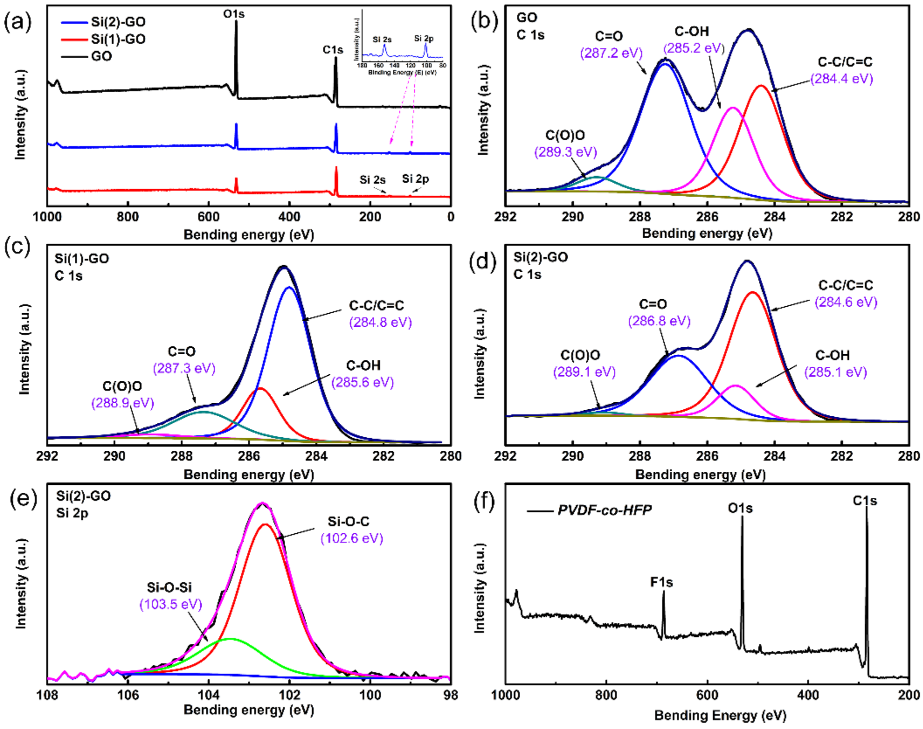

Figure S3. XPS spectra of the Si-modified GO: (**a**) survey scan, (**b**) C1s of GO, (**c**) C1s of Si(1)-GO, (**d**) Si(2)-GO, (**e**) Si 2p of Si(2)-GO; (**f**) survey scan of PVDF-*co*-HFP matrix.

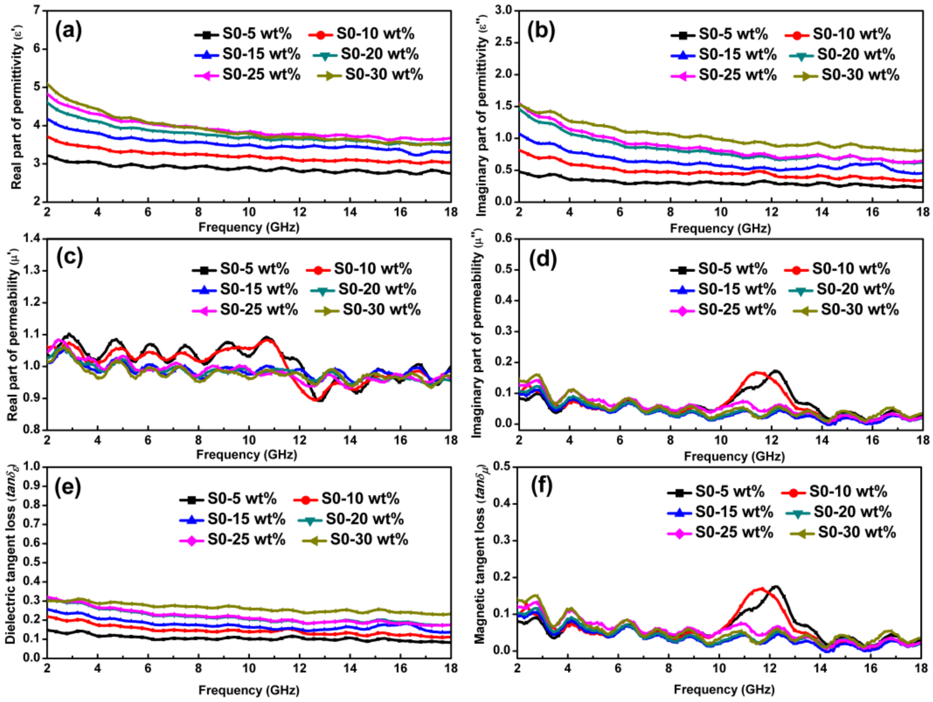

Figure S4. Frequency dependence of the (**a**) real permittivity, (**b**) imaginary permittivity, (**c**) real permeability, (**d**) imaginary permeability, (**e**) dielectric loss, (**f**) magnetic loss of S0 sample with the different mass frations of fillers.

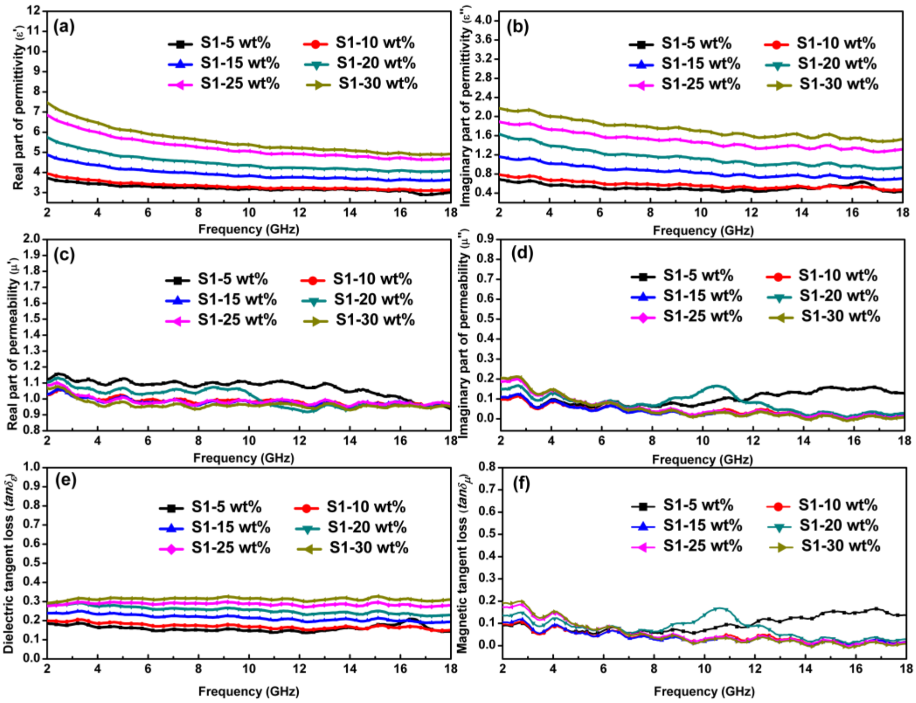

Figure S5. Frequency dependence of the (**a**) real permittivity, (**b**) imaginary permittivity, (**c**) real permeability, (**d**) imaginary permeability, (**e**) dielectric loss, (**f**) magnetic loss of the S1 sample with the different mass fraction of fillers.

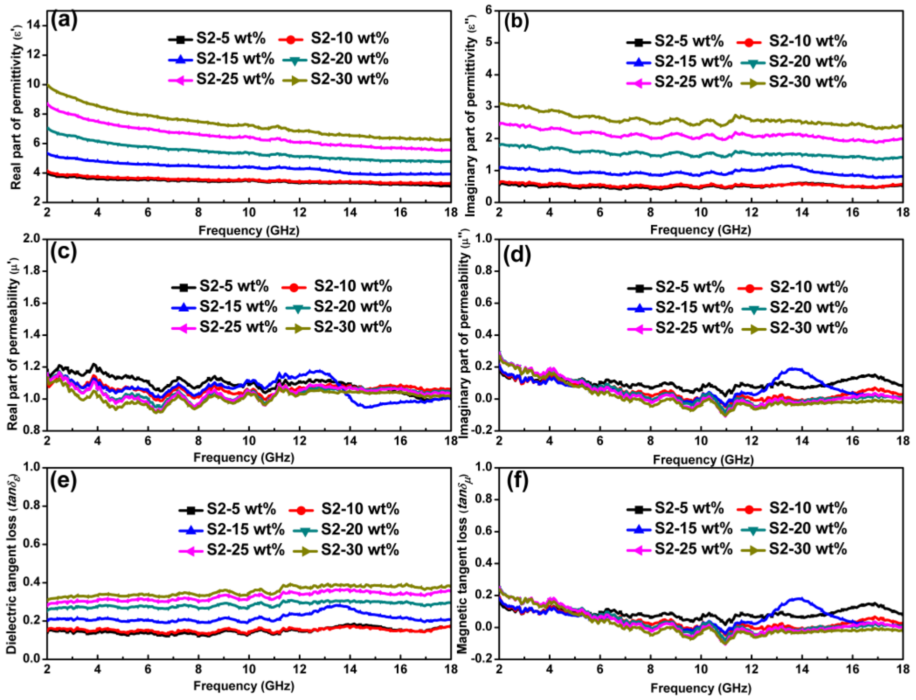

Figure S6. Frequency dependence of the (**a**) real permittivity, (**b**) imaginary permittivity, (**c**) real permeability, (**d**) imaginary permeability, (**e**) dielectric loss, (**f**) magnetic loss of S2 sample with the different mass fraction of fillers.

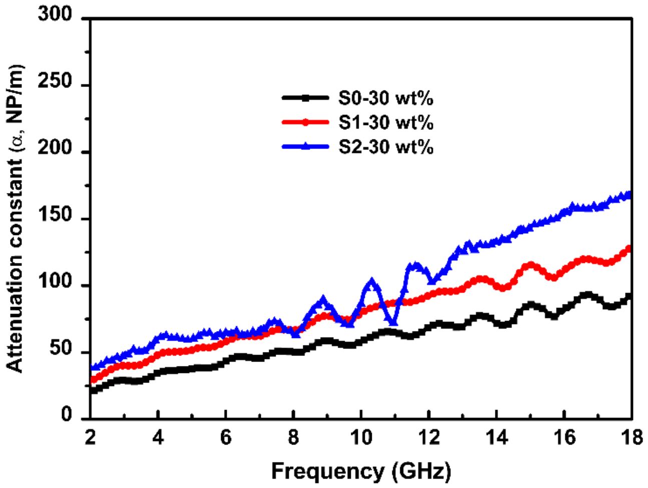

Figure S7. Attenuation constant *α-f* curves of the Si-modified rGO@Fe_3O_4/PVDF-*co*-HFP (S1, S2) composites and rGO@Fe_3O_4/PVDF-*co*-HFP (S0) composite.

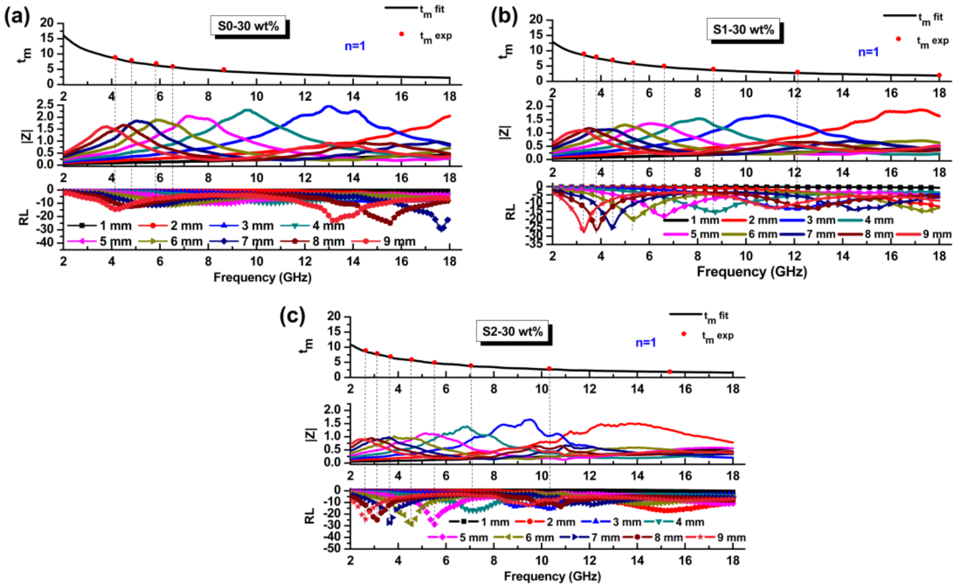

Figure S8. Frequency dependence of the *RL* values, Z modulus (|Z|) and matching thickness (t_m) of samples.

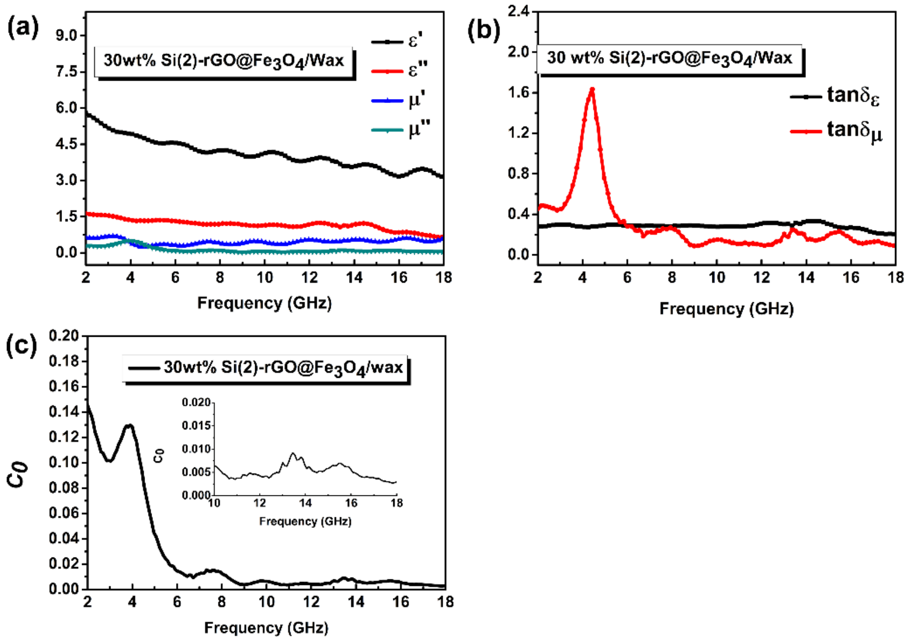

Figure S9. Frequency dependence of the electromagnetic parameters of Si(2)- rGO@Fe_3O_4/paraffin wax composite: (**a**) complex permittivity and permeability, (**b**) complex dielectric loss and magnetic loss, (**c**) C_0 curve.
